# The association between parental postpartum depression and offspring autism spectrum disorder

**DOI:** 10.3389/fpsyt.2025.1693979

**Published:** 2025-11-11

**Authors:** Weiyao Yin, Abraham Reichenberg, Sven Sandin, Michael E. Silverman

**Affiliations:** 1 Department of Medical Epidemiology and Biostatistics, Karolinska Institutet, Stockholm, Sweden; 2 Department of Psychiatry, Icahn School of Medicine at Mount Sinai, New York, NY, United States; 3 Seaver Center for Autism Research and Treatment, Icahn School of Medicine at Mount Sinai, New York, NY, United States; 4 Department of Environmental Medicine and Public Health, Icahn School of Medicine at Mount Sinai, New York, NY, United States

**Keywords:** postpartum depression (PPD), autism spectrum disorder (ASD), healthcare, parental mental health, cohort study, epidemiology

## Abstract

**Objective:**

Postpartum depression (PPD) reportedly affects up to 20% of new mothers. While parental psychiatric history has been associated with an increased likelihood of neurodevelopmental conditions in the offspring, only a few studies of clinically diagnosed PPD exist exploring associated autism spectrum disorder (ASD) outcomes and no study to date has explored the contributions of paternal PPD with ASD risk or the combined influence.

**Methods:**

A nationwide prospective cohort of all live births in Sweden from 1997 through 2021, followed up through December 31, 2022. Associations between parental PPD and ASD were quantified by hazard ratios and two-sided 95% confidence intervals (CIs) from Cox regressions.

**Results:**

Among 1,781,349 live-births, ASD was diagnosed in 986 (4.6%) children of 21,461 born to mothers with PPD (574.3 per 100,000 person-years), 331 (5.3%) of 6,292 born to fathers with PPD (589.0 per 100,000 person-years), and 37 (8.8%) of 420 when both parents had PPD (1177.3 per 100,000 person-years). The hazard ratio of ASD when the mother was diagnosed with PPD was 2.56[CI:2.29-2.85], for fathers 2.59[CI:2.43-2.76] and both 5.54[CI:4.02-7.65]. Adjustment for possible confounders and depression history provided similar trends (mother 1.53[CI:1.36-1.71], fathers 1.71[CI:1.60-1.83] and both 2.19[CI:1.58-3.03]).

**Conclusion:**

Parental PPD was associated with an increased risk of ASD in the offspring, and this association was partially, though not fully, explained by depression history, antidepressant use, and other parental psychiatric factors. The magnitude of the association increased comparably when either parent was diagnosed with PPD and increased further when both parents were diagnosed, with a pattern indicative of shared genetic influences.

## Introduction

Postpartum depression (PPD) reportedly affects 7-20% of new mothers depending largely on ascertainment methods (1). Characterized as a perinatal onset specifier of a unipolar major depressive disorder (2, 3), PPD is unusual among psychiatric disorders in that it is conditionally based on the timing of onset (after childbirth) as opposed to having unique symptomatology. This distinction has led some investigators to question whether PPD is unique compared to other major depressive disorders (4). Indeed, although traditionally attributed to biological factors, genetic, endocrinologic, or immunologic determinants associated with reproduction (5, 6), explorations into the genetic nature of PPD have revealed congruent underpinnings to other unipolar depressive disorders with respect to risk factors (7) and prevalence (8).

Despite significant public health importance, research on PPD and the negative consequences on offspring outcomes (9, 10) has often included important methodological limitations, leaving aspects of the disorder and its impact incompletely characterized. Previous studies of PPD, for example, have primarily relied on symptom inventories rather than clinical diagnoses (11), leading to the overestimation of prevalence (12) and confounding correlations among offspring outcomes. Conversely, studies exploring clinically diagnosed PPD have generally depended on convenience rather than epidemiological samples, which are prone to sampling bias and for which outcomes are not normalizable (13). Finally, depression is understood to be a recurrent disorder, and unsurprisingly large population-based studies (14) and a recent meta-analysis (15) demonstrate that an individual’s depression history explains almost all PPD risk. Yet, most studies of PPD have either ignored or been unable to validate psychiatric history, relying instead on verbal reports, which are prone to bias and recall error thereby making it difficult to quantify the impact of prior psychiatric vulnerabilities. These methodologic limitations have made interpreting the impact of PPD on offspring outcomes challenging. What is therefore required is a systematic and prospective, large-scale, nationally inclusive epidemiological study that examines the impact of clinically diagnosed PPD on childhood outcomes using a complete depression history of both parents as well as outcome measures with complete offspring coverage.

Autism spectrum disorder (ASD) is a chronic neurodevelopmental disorder with high heritability that is generally believed to develop *in utero* secondary to genetic and environmental factors (16). Heterogenous in nature, ASD is defined by deficits in communication and learning, poor social reciprocity, and behavioral abnormalities. Although symptoms of ASD often appear in infancy, ASD is generally not diagnosed before the first two or three years of life (17) and while the causal factors for ASD remain mostly unknown, heredity explains a considerable amount of risk (18). Notably, as ASD has been shown to share genetic etiology with several psychiatric disorders (19, 20), it is unsurprising that studies exploring the association between parental psychiatric conditions whether during gestation (21) or lifetime history (22) and ASD offspring outcomes have observable relationships (23). Unfortunately, most of these earlier studies share limitations akin to the previously noted PPD literature thereby limiting statistical power and diagnostic precision. Recently, a multi-national, population-inclusive study with complete follow-up has highlighted the role of several parental psychiatric disorders in the mother, the father, and both parents *before* birth as risk factors for offspring ASD (20). Consistent with the “polygenic” basis of mental illness risk (24), ASD risk was observed to be high for pre-gestational maternal psychiatric disorders compared to paternal disorders, but highest when both parents had a psychiatric history (20).

The psychosocial adjustment specific to childrearing, *after* birth, and in the early postpartum impacts each parent and, for some, may represent a moment of increased vulnerability for a new or recurrent psychiatric illness, most commonly PPD (1, 4, 5). However, to our knowledge, how maternal or paternal PPD and the combined effect in both parents may relate to the observed risk of ASD outcomes has not yet been prospectively studied. Therefore, this study’s aim is to examine ASD risk in the offspring of parents who received a PPD diagnosis and to examine influences by parental psychiatric and antidepressant medication history, socioeconomic factors, offspring sex, and gestational age as well as changes due to temporal trends of birth year. While PPD may not be etiologically distinct from other depressive disorders (4, 8, 14, 15), its timing, heritability pattern, and developmental context make it an especially informative lens for studying the intergenerational transmission of risk for neurodevelopmental outcomes including ASD (9).

## Methods

### Data sources and study population

All live births in Sweden between January 1, 1997, and December 31, 2021, to parents (both) born in Sweden were assessed. Individual-level data from different Swedish registers was linked via the unique personal identity number of each resident. Biological parents were identified from the Swedish Medical Birth Register (MBR) and the Swedish Multi-generation Register. Vital status and emigration were derived from the Swedish Total Population Register. The study was approved by the National Swedish Ethics Review Board (2017/1875-31/1; 2018/1864-32; 2019-06314). Due to the nature of the study, no individual level consent was required.

### Postpartum depression

In agreement with the Agency for Healthcare Research and Quality, and consistent with prior research, we defined PPD as any diagnoses of PPD, as well as any unipolar depression diagnoses occurring within the first postpartum year (13). The Swedish National Patient Register (NPR) was used to obtain depression diagnoses from specialist care according to the International Classification of Diseases (ICD, v8-v10; inpatient diagnoses since 1973 and outpatient diagnoses since 2001; see **eTable 1** for the included ICD codes). Data quality of the NPR has been verified and validated for diagnoses of multiple psychiatric conditions (25).

PPD was classified as ‘maternal only’ if present only in the gestational parent, ‘paternal only’ if present only in non-gestational parent, or ‘both’ if present in both parents within the first year of delivery, but not necessarily simultaneously (**Table 1**).

**Table 1:** Cohort characteristics by postpartum depression (PPD) in parents.

Characteristics	Neither parent with PPD	Paternal PPD only	Maternal PPD only	Both parents with PPD
	Number of children (%)	Number of children (%)	Number of children (%)	Number of children (%)
Number of individuals	1,753,176 (98.42%)	6,292 (0.35%)	21,461 (1.20%)	420 (0.02%)
Offspring sex (male, %)	901,881 (51.44%)	3,299 (52.43%)	11,091 (51.68%)	220 (52.38%)
Follow-up years*	13.1 (7.3-19.2)	9.3 (5.4-13.8)	8.3 (4.6-12.7)	7.7 (4.8-11.5)
Birth year				
1997-2001	327,525 (18.68%)	215 (3.42%)	568 (2.65%)	4 (0.95%)
2002-2006	360,485 (20.56%)	891 (14.16%)	2,183 (10.17%)	40 (9.52%)
2007-2011	369,773 (21.09%)	1,500 (23.84%)	4,695 (21.88%)	89 (21.19%)
2012-2016	357,620 (20.40%)	1,953 (31.04%)	6,723 (31.33%)	139 (33.10%)
2017-2021	337,773 (19.27%)	1,733 (27.54%)	7,292 (33.98%)	148 (35.24%)
Offspring ASD	49,747 (2.84%)	331 (5.26%)	986 (4.59%)	37 (8.81%)
Autistic	29,554 (59.41%)	250 (75.53%)	721 (73.12%)	31 (83.78%)
Age at ASD*	12.1 (8.4-15.5)	9.7 (6.5-12.5)	9.7 (6.0-12.8)	9.6 (6.4-12.4)
Gestational age				
Preterm <37 week	100,069 (5.71%)	416 (6.61%)	1,794 (8.36%)	24 (5.71%)
Early term 37–38 week	325,045 (18.54%)	1,339 (21.28%)	5,741 (26.75%)	140 (33.33%)
Full term 39+ weeks	1,328,062 (75.75%)	4,537 (72.11%)	13,926 (64.89%)	256 (60.95%)
Parental psychiatric history before delivery				
Maternal depression	66,518 (3.79%)	897 (14.26%)	10,976 (51.14%)	260 (61.90%)
Paternal depression	30,012 (1.71%)	3,211 (51.03%)	1,211 (5.64%)	234 (55.71%)
Maternal depression or ≥ 2 antidepressant records	188,936 (10.78%)	1,984 (31.53%)	16,472 (76.75%)	349 (83.10%)
Paternal depression or ≥ 2 antidepressant records	101,151 (5.77%)	4,452 (70.76%)	3,482 (16.22%)	312 (74.29%)
Maternal any mental illness or ≥ 2 antidepressant records	284,456 (16.23%)	2,600 (41.32%)	17,551 (81.78%)	371 (88.33%)
Paternal any mental illness or ≥ 2 antidepressant records	186,067 (10.61%)	4,958 (78.80%)	5,344 (24.90%)	348 (82.86%)
Maternal age at delivery*	30 (27-34)	30 (26-34)	31 (27-34)	29 (25-34)
Paternal age at delivery*	32 (29-36)	32 (28-37)	32 (29-37)	32 (28-37)
Maternal disposable income (in 100 SEK) in year before delivery*	1,721 (1,228-2,381)	1,735 (1,213-2,352)	1,816 (1,263-2,496)	1,589 (1,069-2,195)
Paternal disposable income (in 100 SEK) in year before delivery*	2,293 (1,624-3,075)	1,864 (1,218-2,596)	2,543 (1,815-3,313)	1,913 (1,240-2,691)
Maternal education				
<9 years primary school	2,328 (0.13%)	39 (0.62%)	87 (0.41%)	7 (1.67%)
9 years primary school	122,493 (6.99%)	1,003 (15.94%)	2,613 (12.18%)	92 (21.90%)
1–2 years secondary school	215,060 (12.27%)	771 (12.25%)	2,222 (10.35%)	61 (14.52%)
3 years secondary school	532,978 (30.40%)	2,154 (34.23%)	6,311 (29.41%)	113 (26.90%)
1–2 years postgraduate	244,360 (13.94%)	688 (10.93%)	2,858 (13.32%)	58 (13.81%)
≥ 3 years postgraduate	624,121 (35.60%)	1,602 (25.46%)	7,227 (33.68%)	85 (20.24%)
PhD	11,836 (0.68%)	35 (0.56%)	143 (0.67%)	4 (0.95%)
Paternal education				
<9 years primary school	4,563 (0.26%)	69 (1.10%)	91 (0.42%)	5 (1.19%)
9 years primary school	159,589 (9.10%)	1,369 (21.76%)	2,492 (11.61%)	105 (25.00%)
1–2 years secondary school	338,554 (19.31%)	1,267 (20.14%)	2,900 (13.51%)	68 (16.19%)
3 years secondary school	568,329 (32.42%)	2,002 (31.82%)	7,827 (36.47%)	138 (32.86%)
1–2 years postgraduate	255,506 (14.57%)	640 (10.17%)	2,891 (13.47%)	50 (11.90%)
≥ 3 years postgraduate	407,825 (23.26%)	914 (14.53%)	5,035 (23.46%)	53 (12.62%)
PhD	18,810 (1.07%)	31 (0.49%)	225 (1.05%)	1 (0.24%)

ASD, autism spectrum disorders; AD, Autistic Disorder; Q1, 1st quartile (25th percentile), Q3, 3rd quartile (75th percentile); SEK, Swedish Krona. *Median (Q1-Q3).

### Outcome: autism spectrum disorder

Diagnoses of ASD from age one onwards were obtained from the NPR using the ICD (**eTable 1**). In Sweden, all infants and preschoolers receive regular medical and developmental evaluations covering motor skills, language, cognitive abilities, and social development. Children suspected of ASD are referred to specialists for assessment. Previous publications document the diagnostic processes and validity of ASD diagnoses in Sweden (26).

### Other covariates

Birth year, offspring sex, maternal age, and gestational age (weeks) data were extracted from the MBR. Paternal age was calculated by linking offspring to their biological fathers through the Multi-Generation Register. Paternal and maternal yearly disposable income (Swedish Krona) and educational attainment (completed school years) were obtained from the Swedish database for health insurance and labor market studies (LISA database). Parental history of antidepressant medication use was defined as at least two dispenses registered in the Swedish Prescribed Drug Register (available since July 2005) using the Anatomical Therapeutic Chemical classification code N06A. Depression and psychiatric history was defined as depression or any psychiatric diagnosis registered in the NPR. All covariates were defined before the birth of the child.

### Statistical analyses

The relative risk of ASD in offspring of parents with PPD compared to offspring of parents without PPD was quantified by hazard ratios (HR) and associated two-sided 95% confidence intervals (CI) from Cox proportional hazards models, together with ASD incidence (cases per 100,000 person-years). The age at follow-up was the underlying time-scale. Offspring were followed from age one until ASD diagnosis, emigration, death, or December 31, 2022, whichever came first.

All statistical models were pre-specified before performing the analysis and based on prior literature (20). In Model 1 PPD in fathers, mothers, and both parents were adjusted for offspring birth year using natural cubic splines with five internal knots. Parental age (years), education (< 9 years primary education, 9 years primary education, 1-2 years secondary education, 3 years secondary education, 1-2 years postgraduate education, ≥ 3 years postgraduate education, PhD education), income (ordered into yearly ventiles), and preterm birth (yes/no) were additionally adjusted for in Model 2 to account for potential influences. Continuous variables were modeled using natural cubic splines (20). Parental depression diagnoses assigned before childbirth was additionally adjusted for in Model 3. Model 4 expanded Model 3 to include a history of >2 dispenses of antidepressant prescriptions, and Model 5 expanded Model 4 to adjust for any parental psychiatric diagnosis assigned before childbirth.

The proportional hazards assumption of the Cox regression was visually examined by weighted Schoenfeld residuals. Statistical tests were performed on the two-sided 5% level of significance. P-values were not adjusted for multiplicity of statistical tests, however, the primary research question is composed of only three statistical tests (PPD in the mother, the father, and in both parents, compared to neither parent with PPD). To address potential correlation between siblings, robust standard errors were applied.

### Supplementary analyses

Psychiatric disorders before childbirth are associated with higher risk of preterm delivery (27), and increased risk of ASD in offspring (20). Whether ASD risk was modified by gestational age was explored by including an interaction term between PPD and gestational age (preterm term <37 weeks, early-term 37–38 weeks, full-term >39 weeks). ASD risk was also estimated separately by offspring sex. Because earlier PPD onset may represent a distinct etiological group, PPD was restricted to the first six weeks postpartum. To identify potential patients who seek help only in primary care, we expanded the definition of PPD to include those with either a depression diagnosis or the record of an antidepressant prescription during the postpartum period. We analyzed cases where PPD was the first instance of depression versus a recurrence. For first-ever depression, we included children of parents without prior depression or fewer than two antidepressant records before delivery. For recurrence depression, we included children of either parent with a depression history or at least two antidepressant records before delivery. We separately examined parents with an antidepressant prescription within one year before delivery. Due to the potential unreliability of ASD diagnoses before age two, we only considered ASD diagnoses after age two, and followed up with all children from age two onwards. Parents with ASD were excluded to test whether this risk was driven by the inherited ASD risk from parents.

The SAS software was used for all data management work and statistical analyses (Cox regression using Proc PHREG).

## Results

The study population included 1,794,593 births in Sweden to Swedish-born parents between 1997 and 2021. Of these, 5,031 (0.28%) were excluded from the analysis due to death, and emigration prior to age one, and 8,213 (0.46%) were removed due to incomplete covariate data. The analytic cohort included 1,781,349 children (916,491 male, 864,858 female), contributing 21,670,307 person-years during an average follow-up of 12 years. In total, 6,292 (0.4%) of fathers only, 21,461 (1.2%) of mothers only, and 420 (0.02%) of both parents had a PPD diagnosis. Among 1,753,176 parents without a PPD diagnosis, 49,747 (2.8%) children had ASD (232 ASD per 100,000 person-years). In comparison, 331 (5.3%) children had a diagnosis for ASD (589 ASD per 200,000 person-years) when only the father was diagnosed with PPD, 986 (4.6%) children had ASD (574 ASD per 100,000 person-years) when only the mother was diagnosed with PPD, and 37 (8.8%) children had ASD (1177 ASD per 100,000 person-years) when both parents had PPD. For mothers diagnosed with PPD, the HR of an ASD outcome was 2.59[CI:2.43-2.76], for fathers 2.56[CI: 2.29-2.85], and both parents 5.54[CI: 4.02-7.65] (**Table 2**, Model 1). When parental depression history, parental age, education, income, and preterm delivery were controlled the HR of an ASD diagnosis when the mother was diagnosed with PPD was 1.71[CI:1.60-1.83], for fathers 1.53[CI:1.36-1.71] and both 2.19[CI:1.58-3.03] (**Table 2**, Model 3). Further adjustment for parental antidepressant use (**Table 2**, Model 4), followed by additional adjustment for any psychiatric history and pre-delivery antidepressant use (**Table 2**, Model 5) did not further reduce the magnitude of the association. No evidence for non-proportional hazards was detected (**eFigure 1**).

**Table 2:** Parental postpartum depression and relative risk of ASD in offspring.

Postpartum depression	Total subjects	Person-years	ASD (rate)	Model 1	Model 2	Model 3	Model 4	Model 5
Neither parent	1,753,176	21,439,288	49,747 (232.0)	Reference	Reference	Reference	Reference	Reference
Fathers only	6,292	56,192	331 (589.0)	2.56 (2.29-2.85)	1.87 (1.68-2.09)	1.53 (1.36-1.71)	1.43 (1.28-1.60)	1.42 (1.27-1.59)
Mothers only	21,461	171,684	986 (574.3)	2.59 (2.43-2.76)	2.21 (2.08-2.36)	1.71 (1.60-1.83)	1.56 (1.46-1.67)	1.61 (1.51-1.72)
Both parents	420	3,143	37 (1177.3)	5.54 (4.02-7.65)	3.56 (2.58-4.92)	2.19 (1.58-3.03)	2.01 (1.45-2.77)	2.14 (1.55-2.95)

ASD, Autism Spectrum Disorders.

Postpartum depression: any depression diagnosed in the first year after delivery.

Hazard Ratios and 95% confidence intervals, as estimates of relative risk of ASD in offspring of parents with postpartum depression compared to offspring of parents without postpartum depression, were calculated from Cox regression models, using age at follow-up as the underlying time scale, with robust standard errors. Incidence rate of ASD per 100,000 person years. Followed up all children from age one.

Model 1: Adjusted for birth year by cubic natural splines with five internal knots.

Model 2: Adjusted for birth year, preterm birth (yes/no), maternal and paternal age (years; as natural cubic splines), yearly income (modelled by ranks as natural cubic splines), and highest educational attainment (< 9 years of primary education, 9 years of primary education, 1-2 years of secondary school education, 3 years of secondary school education, 1-2 years of postgraduate education, ≥ 3 years of postgraduate education, PhD education), all defined at delivery.

Model 3: Adjusted for birth year, preterm birth, maternal and paternal age, income, highest educational attainment, and any parental diagnosis of depression before delivery (yes/no).

Model 4: Adjusted for birth year, preterm birth, maternal and paternal age, income, highest educational attainment, and any parental diagnosis of depression or at least 2 records of antidepressant prescriptions before delivery (yes/no).

Model 5: Adjusted for birth year, preterm birth, maternal and paternal age, income, highest educational attainment, and any parental diagnosis of mental illness or at least 2 records of antidepressant prescriptions before delivery (yes/no).

## Supplementary results

There was no support for modification by gestational age (**Table 3**) or offspring birth sex (**Table 4**). The timing of PPD, before or after 6 weeks post-delivery, did not influence the association with ASD (**Table 5**). When broadening the definition of PPD to include any diagnosis of depression or antidepressant prescription during the postpartum period, a total of 143,129 children were identified as having been exposed to parental PPD (when PPD was defined strictly through clinical depression diagnoses, 28,173 children were exposed). In models where history of depression and antidepressant prescription were adjusted for, the association between maternal PPD and offspring ASD remained comparable whereas the paternal association slightly declined (**eTable 2**). The strength of the association was similar when the PPD episode was a first depression or a recurrence (**eTables 3, 4**), and among parents who had been prescribed antidepressants within the year preceding childbirth (**eTable 5**). Furthermore, the risk of ASD was similar when children were followed up from age two (**eTable 6**), or after excluding parents with ASD diagnosis (**eTable 7**).

**Table 3:** Parental postpartum depression and relative risk of ASD in offspring by preterm, early term and full-term births.

Gestational age categories	Postpartum depression	Total subjects	Person-years	ASD (rate)	Model 1	Model 2	Model 3
Preterm birth	Neither parent	100,069	1,249,079	4,069 (325.8)	Reference	Reference	Reference
<37 weeks	Fathers only	416	3,689	26 (704.7)	2.22 (1.51-3.27)	1.64 (1.12-2.42)	1.39 (0.94-2.04)
	Mothers only	1,794	14,59	108 (740.2)	2.37 (1.96-2.87)	2.00 (1.65-2.42)	1.51 (1.24-1.83)
	Both parents	24	173	2 (1159.4)	3.95 (0.99-15.80)	2.38 (0.59-9.51)	1.43 (0.36-5.74)
Early term	Neither parent	325,045	3,985,836	10,070 (252.6)	Reference	Reference	Reference
37–38 weeks	Fathers only	1,339	11,792	87 (737.8)	2.94 (2.38-3.64)	2.14 (1.73-2.65)	1.73 (1.40-2.14)
	Mothers only	5,741	45,103	297 (658.5)	2.75 (2.45-3.09)	2.39 (2.13-2.68)	1.79 (1.59-2.02)
	Both parents	140	1,093	18 (1647.4)	7.19 (4.53-11.42)	4.86 (3.06-7.72)	2.97 (1.87-4.73)
Full term	Neither parent	1,328,062	16,204,373	35,608 (219.7)	Reference	Reference	Reference
≥ 39 weeks	Fathers only	4,537	40,711	218 (535.5)	2.45 (2.14-2.80)	1.80 (1.58-2.06)	1.48 (1.29-1.69)
	Mothers only	13,926	111,991	581 (518.8)	2.46 (2.26-2.67)	2.15 (1.98-2.33)	1.67 (1.53-1.81)
	Both parents	256	1,878	17 (905.4)	4.47 (2.78-7.19)	2.83 (1.76-4.55)	1.77 (1.10-2.86)

ASD, Autism Spectrum Disorders.

Postpartum depression: any depression diagnosed in the first year after delivery.

Hazard Ratios and 95% confidence intervals, as estimates of relative risk of ASD in offspring of parents with postpartum depression compared to offspring of parents without postpartum depression, were calculated from Cox regression models, using age at follow-up as the underlying time scale, including interaction between parental postpartum depression and gestational age categories, with robust standard errors. Incidence rate of ASD per 100,000 person years. Followed up all children from age one. Strata-specific effect is shown. Test for interaction term postpartum history*gestational age categories p=0.32.

Model 1: Adjusted for birth year by cubic natural splines with five internal knots.

Model 2: Adjusted for birth year, maternal and paternal age (years; as natural cubic splines), yearly income (modelled by ranks as natural cubic splines), and highest educational attainment (< 9 years of primary education, 9 years of primary education, 1-2 years of secondary school education, 3 years of secondary school education, 1-2 years of postgraduate education, ≥ 3 years of postgraduate education, PhD education), all defined at delivery.

Model 3: Adjusted for birth year, maternal and paternal age, income, highest educational attainment, and any parental diagnosis of depression before delivery (yes/no).

**Table 4:** Parental postpartum depression and relative risk of ASD in offspring by sex.

Sex	Postpartum depression	Total subjects	Person-years	ASD (rate)	Model 1	Model 2	Model 3
Male	Neither parent	901,881	10,986,531	31,949 (290.8)	Reference	Reference	Reference
	Fathers only	3,299	28,965	220 (759.5)	2.64 (2.32-3.02)	1.96 (1.72-2.24)	1.60 (1.39-1.83)
	Mothers only	11,091	88,389	655 (741.0)	2.67 (2.47-2.89)	2.30 (2.13-2.49)	1.76 (1.62-1.91)
	Both parents	220	1,622	21 (1294.6)	4.91 (3.21-7.52)	3.19 (2.08-4.87)	1.91 (1.24-2.93)
Female	Neither parent	851,295	10,452,757	17,798 (170.3)	Reference	Reference	Reference
	Fathers only	2,993	27,227	111 (407.7)	2.40 (1.99-2.89)	1.73 (1.43-2.08)	1.41 (1.16-1.70)
	Mothers only	10,370	83,295	331 (397.4)	2.43 (2.18-2.71)	2.07 (1.86-2.31)	1.58 (1.41-1.76)
	Both parents	200	1,521	16 (1052.2)	6.74 (4.13-11.01)	4.30 (2.63-7.03)	2.66 (1.63-4.36)

ASD, Autism Spectrum Disorders.

Hazard Ratios and 95% confidence intervals, as estimates of relative risk of ASD in offspring of parents with postpartum depression compared to offspring of parents without postpartum depression, were calculated from Cox regression models, using age at follow-up as the underlying time scale, including interaction between parental postpartum depression and offspring sex, with robust standard errors. Incidence rate of ASD per 100,000 person years. Followed up all children from age one. Strata-specific effect is shown. Test for interaction term postpartum depression*sex p=0.19.

Model 1: Adjusted for birth year by cubic natural splines with five internal knots.

Model 2: Adjusted for birth year, preterm birth (yes/no), maternal and paternal age (years; as natural cubic splines), yearly income (modelled by ranks as natural cubic splines), and highest educational attainment (< 9 years of primary education, 9 years of primary education, 1-2 years of secondary school education, 3 years of secondary school education, 1-2 years of postgraduate education, ≥ 3 years of postgraduate education, PhD education), all defined at delivery.

Model 3: Adjusted for birth year, preterm birth, maternal and paternal age, income, highest educational attainment, and any parental diagnosis of depression before delivery (yes/no).

**Table 5:** Parental postpartum depression within six weeks after delivery and relative risk of ASD in offspring.

Postpartum depression	Total subjects	Person-years	ASD (rate)	Model 1	Model 2	Model 3	Model 4	Model 5
Neither parent	1,769,600	21,587,810	50,638 (234.6)	Reference	Reference	Reference	Reference	Reference
Fathers only	1,034	8,698	55 (632.3)	2.73 (2.10-3.56)	2.00 (1.54-2.61)	1.41 (1.08-1.84)	1.37 (1.05-1.78)	1.41 (1.08-1.83)
Mothers only	10,680	73,579	404 (549.1)	2.59 (2.35-2.86)	2.31 (2.09-2.54)	1.66 (1.50-1.83)	1.48 (1.34-1.63)	1.58 (1.43-1.75)
Both parents	35	219	4 (1823.8)	9.64 (3.67-25.31)	7.10 (2.72-18.56)	3.82 (1.43-10.14)	3.23 (1.21-8.59)	3.60 (1.35-9.59)

ASD, Autism Spectrum Disorders.

Postpartum depression: any depression diagnosed within six weeks after delivery.

Hazard Ratios with 95% confidence intervals from Cox regression models, using age at follow-up as the underlying time scale, with robust standard errors. Incidence rate of ASD per 100,000 person years. Followed up all children from age one.

Model 1: Adjusted for birth year by cubic natural splines with five internal knots.

Model 2: Adjusted for birth year, preterm birth (yes/no), maternal and paternal age (years; as natural cubic splines), yearly income (modelled by ranks as natural cubic splines), and highest educational attainment (< 9 years of primary education, 9 years of primary education, 1-2 years of secondary school education, 3 years of secondary school education, 1-2 years of postgraduate education, ≥ 3 years of postgraduate education, PhD education), all defined at delivery.

Model 3: Adjusted for birth year, preterm birth, maternal and paternal age, income, highest educational attainment, and any parental diagnosis of depression before delivery (yes/no).

Model 4: Adjusted for birth year, preterm birth, maternal and paternal age, income, highest educational attainment, and any parental diagnosis of depression or at least 2 records of antidepressant prescriptions before delivery (yes/no).

Model 5: Adjusted for birth year, preterm birth, maternal and paternal age, income, highest educational attainment, and any parental diagnosis of mental illness or at least 2 records of antidepressant prescriptions before delivery (yes/no).

## Discussion

Using the largest population-inclusive sample to date of clinically diagnosed PPD with psychiatric and offspring follow-up, this longitudinal study examined the relationship between PPD in either or both parents and the risk of ASD in the offspring. The magnitude of association between PPD and ASD in the offspring was similarly increased if either of the parents was diagnosed with PPD, and increased even further if both parents were diagnosed. After adjusting separately for parental depression history, antidepressant use, or any psychiatric history before delivery, this association was partially reduced.

Having a newborn is a stressful experience for most, if not all, new parents, and depressive symptomatology in the postpartum period is a regularly observed occurrence that may affect either parent. However, because diagnostic symptoms common to depression overlap with the normal early adjustment to the childrearing experience, studies limited to symptom inventories alone grossly overestimate PPD prevalence (12), whereas the incidence of clinically significant depression diagnoses in the postpartum are much less common (13). Interestingly, because depression is understood to be a recurrent disorder, it is not surprising that large epidemiological studies of PPD demonstrate that a depression history explains most risk (13, 28). By the same token, a unique pathogenesis of PPD in relation to other unipolar depressive disorders has yet to be established (4, 29).

The time surrounding birth is a critical time for early developmental events and parental depression has been linked to adverse consequences for the offspring including impaired cognitive and language development (30), poor adjustment (31), behavioral problems (32), growth delay (33), fine motor skill (34), and neurodevelopmental challenges (35). However, whether these adverse outcomes are specific to PPD, or genetic factors associated with the parent’s depression or other psychiatric history remains unknown. Indeed, while genetic factors likely play a role in the development of some of these outcomes, non-genetic (environmental) risk factors and interactions between environment and genetics likely also contribute to some of these risks.

ASD represents a heterogeneous neurodevelopmental syndrome with deficits that fall into core domains and begin to emerge in early infancy (17). The genesis of ASD is believed to have both biological and environmental underpinnings with a shared genetic and metabolic etiology common to other psychiatric disorders including schizophrenia, bipolar disorder, and major depressive disorder (36). At the same time, genes inherited from parents are considered the major pathway for ASD; however given the numerous factors associated with healthy brain development, critical knowledge gaps remain. Indeed, an in-depth understanding of how ASD risk varies according to individual parent or total parental contributions (such as, genetic load), and how the various comorbid psychiatric histories may further inform ASD risk remains to be fully elucidated.

Although several large studies have examined the relationship between maternal PPD and ASD in offspring (35, 37, 38), and one has included both parents (22), none have done so prospectively. This is the first study to prospectively examine maternal, paternal, and combined parental PPD in relation to offspring ASD and PPD while also accounting for a broad range of covariates, including maternal and paternal autism and autism traits, disposable income, medication exposure, and educational attainment. Notably, while an association was observed between maternal PPD and ASD in the offspring, comparable to that found in the Australian population (38), paternal PPD was also found to be associated with increased ASD risk and greatest when both parents were diagnosed with PPD – similar to a pattern observed in Taiwan (22). This finding is also consistent with findings from Swedish and Finnish populations exploring the association of a wide array of pre-delivery psychiatric disorders with the risk of ASD in the offspring (20). The observation of increased ASD risk when only the father is diagnosed, and with the highest risk when both parents are diagnosed with PPD, may reflect a shared genetic etiology. That the combined effect of maternal and paternal PPD on ASD outcomes is additive, suggests that common genetic variants may be inherited from both parents (39). However, that this combined risk reduced when depression history in the parent was controlled may help inform future studies exploring underlying genetic and environmental mechanisms. Notably, although the relative risk increased two-fold to five-fold for children born to parents where both parents were diagnosed with PPD, the clinical implications and public impact are limited given the rarity of this observation.

The time surrounding childbirth is a time of significant vulnerability for the recurrence of various psychiatric disorders in the parents (40, 41). Importantly, although the timing of depression onset within the postpartum period may appear to be indicative of a biological etiology associated with the reproductive process, an earlier study exploring whether the period after childbirth was associated with depression revealed no differences in risk compared to a period following a randomly generated date (8). In this respect, studies into heritability and familial aggregation have pointed to PPD as being linked to genetic etiologies of unipolar depression, unrelated to reproduction, as well as polygenic risk scores nonspecific to childbirth (42). In the present study, after controlling for depression history, antidepressant use, and mental illness before delivery, the association between PPD and ASD remained. This suggests that a history of depression alone may not account for the association between parental psychopathology in the postpartum period and ASD. It may instead indicate that PPD occurring in parents without a prior history of depression reflects an enduring vulnerability to depressive illness which may have been triggered by the uniquely stressful conditions of early childrearing. However, it is equally important to note that because postpartum women are a medically captured population in Sweden available for PPD screening, the rate of treatment-seeking behaviors for a depressive illness before pregnancy is likely incomplete (43). In this respect, the observed association between PPD and ASD in the context of a psychiatric history and SSRI treatments likely represents an underestimation. Similarly, while the resolution of our data does not allow for further exploration of potential underlying influences, it would be inappropriate to imply that PPD causes ASD particularly given that PPD occurs after pregnancy, whereas ASD is thought to likely develop during the prenatal period (44, 45). Therefore, the observed association between PPD and ASD likely reflects shared genetic influences (pleiotropy), which may represent potential biological mechanisms (46). A genetic link between PPD and ASD is further supported by the observation of an increased risk of ASD in children born to fathers with PPD who are free from the biological factors specific to pregnancy and delivery.

This study comes with many strengths, including the large sample size and prospective design. The use of prospectively collected and clinically ascertained diagnoses of PPD/depression and ASD from within a publicly financed and universal health system with national coverage minimizes a wide range of potential biases. The extensive database and longitudinal design allow for detailed adjustment for potential confounding. Sweden’s public health system includes maternal care during and after pregnancy, including postpartum “in-home” visits with nearly 100% participation (47). This reduces the selection bias from a group of individuals with a higher frequency of treatment-seeking behavior.

The study also has limitations. The NPR does not include diagnoses from primary care. However, the availability of prescribed drug data captures those individuals treated in primary care who were prescribed antidepressants, and these cases were included in our analyses. We were not able to capture those with depression who do not visit health care and those who visit primary care but do not receive medications. We lacked information to distinguish between children raised by non-biological parents, but in Sweden, this accounts for only a small proportion of children (48). Because ASD diagnoses are only available in the ICD-9 and ICD-10 in the Swedish register, parents with ASD diagnosed exclusively prior to 1987 would not have been captured, and thus there remains a possibility of residual confounding from undetected parental ASD. Genetic confounding may also have occurred if parents with PPD are a specific group with a higher inherited risk of ASD. However, this genetic confounding could not fully account for the remaining association, because the results did not change after excluding parents diagnosed with ASD. Finally, as in any epidemiological study, we cannot rule out bias due to residual and unmeasured confounding. Therefore, our findings should be replicated in other populations and healthcare systems.

## Conclusion

Using the largest nationally inclusive sample to date with clinically ascertained ASD and depression diagnoses, PPD was associated with an increased risk of ASD in the offspring, and this association was partially, though not fully, explained by depression history, antidepressant use, and other parental psychiatric factors. The magnitude of the association increased comparably when either parent was diagnosed with PPD and increased further when both parents were diagnosed - a pattern indicative of shared genetic influences.

## Data Availability

The datasets generated and analyzed during the current study are not publicly available due to patient protections, institutional and state policy. Data are available from the Swedish Government upon request to the Swedish Registers. Requests to access the datasets should be directed to patientregistret@socailstyrelsen.se.
