# Supplementary material for: The association between parental postpartum depression and offspring autism spectrum disorder
Source: Front Psychiatry. 2025 Nov 11;16:1693979. doi: 10.3389/fpsyt.2025.1693979 (PMC12645387; doi:10.3389/fpsyt.2025.1693979)
Supplement: Supplementary file 1 [file Supplementaryfile1.pdf]

Weiyao Yin, Abraham Reichenberg, Sven Sandin, Michael E. Silverman

Supplementary Methods, Tables and Figures

List of contents

eTables

eTable 1 ICD codes for psychiatric disorders 2

eTable 2 Diagnosis of depression or a record of antidepressant prescription during postpartum period in parents and risk of ASD in offspring 3

eTable 3 Association between parental postpartum depression and offspring ASD risk among parents without prior depression or fewer than two antidepressant prescriptions before delivery 4

eTable 4 Association between parental postpartum depression and offspring ASD risk when either parent has a depression history or at least two antidepressant prescriptions before delivery.................5

eTable 5 Association between parental postpartum depression and offspring ASD risk when either parent has an antidepressant prescription within one year before delivery 6

eTable 6 Association between parental postpartum depression and offspring ASD risk followed up from age two onwards 7

eTable 7 Association between parental postpartum depression and offspring ASD risk among parents without an ASD diagnosis before delivery 8

eFigures

eFigure 1 Weighted Schoenfeld residual plot to assess proportional hazards assumption.........................9

eTable 1 ICD codes for psychiatric disorders

Disorder	ICD-10	ICD-9	ICD-8
Overall mental illness	F10-F99	291, 295, 296, 297, 298, 299, 300, 301, 302, 303, 304, 305, 306, 307, 308, 309, 311, 312, 313, 314, 315, 316, 317, 318, 319	291, 295, 296, 297, 298, 299, 300, 301, 302, 303, 304, 305, 306, 307, 308, 310, 311, 312, 313, 314, 315
Depression	F32, F33, F341, F348, F349, F530	296B, 300E, 311, 301B	296.0, 298.0, 300.4, 301.1
ASD	F840, F841, F843, F845, F848, F849	299A	

eTable 2 Diagnosis of depression or a record of antidepressant prescription during postpartum period in parents and risk of ASD in offspring

Postpartum depression	Total subjects	Person-years	ASD (rate)	Model 1	Model 2	Model 3	Model 4	Model 5
Neither parent	1,638,220	20,601,319	46,191 (224.2)	Reference	Reference	Reference	Reference	Reference
Fathers only	47,858	381,898	1,467 (384.1)	1.78 (1.69-1.87)	1.52 (1.44-1.60)	1.42 (1.35-1.50)	1.25 (1.18-1.33)	1.21 (1.14-1.28)
Mothers only	84,306	614,310	2,954 (480.9)	2.34 (2.25-2.43)	2.03 (1.95-2.11)	1.79 (1.72-1.87)	1.50 (1.43-1.56)	1.52 (1.46-1.58)
Both parents	10,965	72,780	489 (671.9)	3.44 (3.14-3.76)	2.56 (2.34-2.81)	2.08 (1.90-2.29)	1.55 (1.41-1.71)	1.58 (1.44-1.73)

Abbreviations. ASD: Autism Spectrum Disorders.

Postpartum depression: any depression diagnosed in the first year after delivery.

Hazard Ratios with 95% confidence intervals from Cox regression models, using age at follow-up as the underlying time scale, with robust standard errors. Incidence rate of ASD per 100,000 person years. Followed up all children from age one.

Model 1: Adjusted for birth year by cubic natural splines with five internal knots;

Model 2: Adjusted for birth year, preterm birth (yes/no), maternal and paternal age (years; as natural cubic splines), yearly income (modelled by ranks as natural cubic splines), and highest educational attainment (< 9 years of primary education, 9 years of primary education, 1-2 years of secondary school education, 3 years of secondary school education, 1-2 years of postgraduate education, ≥ 3 years of postgraduate education, PhD education), all defined at delivery.

Model 3: Adjusted for birth year, preterm birth, maternal and paternal age, income, highest educational attainment, and any parental diagnosis of depression before delivery (yes/no).

Model 4: Adjusted for birth year, preterm birth, maternal and paternal age, income, highest educational attainment, and any parental diagnosis of depression or at least 2 records of antidepressant prescriptions before delivery (yes/no).

Model 5: Adjusted for birth year, preterm birth, maternal and paternal age, income, highest educational attainment, and any parental diagnosis of mental illness or at least 2 records of antidepressant prescriptions before delivery (yes/no).

eTable 3 Association between parental postpartum depression and offspring ASD risk among parents without prior depression or fewer than two antidepressant prescriptions before delivery

Postpartum depression	Total subjects	Person-years	ASD (rate)	Model 1	Model 2
Neither parent	1,493,406	19,666,196	42,876 (218.0)	Reference	Reference
Fathers only	1,501	18,649	102 (546.9)	2.38 (1.96-2.90)	1.78 (1.47-2.16)
Mothers only	4,571	54,932	265 (482.4)	2.14 (1.89-2.41)	1.86 (1.64-2.09)
Both parents	33	387	4 (1033.9)	4.65 (1.76-12.29)	3.12 (1.18-8.20)

Abbreviations. ASD: Autism Spectrum Disorders.

Postpartum depression: any depression diagnosed in the first year after delivery.

Hazard Ratios with 95% confidence intervals from Cox regression models, using age at follow-up as the underlying time scale, with robust standard errors. Incidence rate of ASD per 100,000 person years. Followed up all children from age one.

Model 1: Adjusted for birth year by cubic natural splines with five internal knots;

Model 2: Adjusted for birth year, preterm birth (yes/no), maternal and paternal age (years; as natural cubic splines), yearly income (modelled by ranks as natural cubic splines), and highest educational attainment (< 9 years of primary education, 9 years of primary education, 1-2 years of secondary school education, 3 years of secondary school education, 1-2 years of postgraduate education, ≥ 3 years of postgraduate education, PhD education), all defined at delivery.

eTable 4 Association between parental postpartum depression and offspring ASD risk when either parent has a depression history or at least two antidepressant prescriptions before delivery

Postpartum depression	Total subjects	Person-years	ASD (rate)	Model 1	Model 2	Model 3	Model 4	Model 5
Neither parent	259,770	1,773,092	6,871 (387.5)	Reference	Reference	Reference	Reference	Reference
Fathers only	4,791	37,543	229 (610.0)	1.46 (1.28-1.67)	1.31 (1.14-1.49)	1.31 (1.14-1.50)	1.33 (1.16-1.52)	1.34 (1.18-1.54)
Mothers only	16,890	116,752	721 (617.5)	1.58 (1.46-1.71)	1.56 (1.44-1.68)	1.45 (1.34-1.57)	1.44 (1.34-1.56)	1.46 (1.35-1.57)
Both parents	387	2,756	33 (1197.4)	3.10 (2.21-4.37)	2.54 (1.80-3.57)	2.30 (1.63-3.24)	2.05 (1.45-2.89)	2.15 (1.52-3.02)

Abbreviations. ASD: Autism Spectrum Disorders.

Hazard Ratios with 95% confidence intervals from Cox regression models, using age at follow-up as the underlying time scale, with robust standard errors. Incidence rate of ASD per 100,000 person years. Followed up all children from age one.

Model 1: Adjusted for birth year by cubic natural splines with five internal knots;

Model 2: Adjusted for birth year, preterm birth (yes/no), maternal and paternal age (years; as natural cubic splines), yearly income (modelled by ranks as natural cubic splines), and highest educational attainment (< 9 years of primary education, 9 years of primary education, 1-2 years of secondary school education, 3 years of secondary school education, 1-2 years of postgraduate education, ≥ 3 years of postgraduate education, PhD education), all defined at delivery.

Model 3: Adjusted for birth year, preterm birth, maternal and paternal age, income, highest educational attainment, and any parental diagnosis of depression before delivery (yes/no).

Model 4: Adjusted for birth year, preterm birth, maternal and paternal age, income, highest educational attainment, and any parental diagnosis of depression or at least 2 records of antidepressant prescriptions before delivery (yes/no).

Model 5: Adjusted for birth year, preterm birth, maternal and paternal age, income, highest educational attainment, and any parental diagnosis of mental illness or at least 2 records of antidepressant prescriptions before delivery (yes/no).

eTable 5 Association between parental postpartum depression and offspring ASD risk when either parent has an antidepressant prescription within one year before delivery

Postpartum depression	Total subjects	Person-years	ASD (rate)	Model 1	Model 2	Model 3	Model 4	Model 5
Neither parent	102,745	713,132	2,998 (420.4)	Reference	Reference	Reference	Reference	Reference
Fathers only	3,420	25,289	153 (605.0)	1.43 (1.22-1.68)	1.29 (1.10-1.52)	1.29 (1.08-1.53)	1.32 (1.12-1.56)	1.32 (1.12-1.56)
Mothers only	12,487	81,125	492 (606.5)	1.53 (1.39-1.69)	1.52 (1.38-1.67)	1.33 (1.21-1.48)	1.36 (1.23-1.50)	1.37 (1.25-1.51)
Both parents	324	2,189	25 (1142.3)	2.91 (1.96-4.31)	2.42 (1.63-3.59)	2.07 (1.39-3.09)	1.88 (1.26-2.79)	1.96 (1.32-2.91)

Abbreviations. ASD: Autism Spectrum Disorders.

Hazard Ratios with 95% confidence intervals from Cox regression models, using age at follow-up as the underlying time scale, with robust standard errors. Incidence rate of ASD per 100,000 person years. Followed up all children from age one.

Model 1: Adjusted for birth year by cubic natural splines with five internal knots;

Model 2: Adjusted for birth year, preterm birth (yes/no), maternal and paternal age (years; as natural cubic splines), yearly income (modelled by ranks as natural cubic splines), and highest educational attainment (< 9 years of primary education, 9 years of primary education, 1-2 years of secondary school education, 3 years of secondary school education, 1-2 years of postgraduate education, ≥ 3 years of postgraduate education, PhD education), all defined at delivery.

Model 3: Adjusted for birth year, preterm birth, maternal and paternal age, income, highest educational attainment, and any parental diagnosis of depression before delivery (yes/no).

Model 4: Adjusted for birth year, preterm birth, maternal and paternal age, income, highest educational attainment, and any parental diagnosis of depression or at least 2 records of antidepressant prescriptions before delivery (yes/no).

Model 5: Adjusted for birth year, preterm birth, maternal and paternal age, income, highest educational attainment, and any parental diagnosis of mental illness or at least 2 records of antidepressant prescriptions before delivery (yes/no).

eTable 6 Association between parental postpartum depression and offspring ASD risk followed up from age two onwards

Postpartum depression	Total subjects	Person-years	ASD (rate)	Model 1	Model 2	Model 3
Neither parent	1,683,402	19,718,394	49,630 (251.7)	Reference	Reference	Reference
Fathers only	5,989	50,053	330 (659.3)	2.56 (2.29-2.85)	1.87 (1.68-2.09)	1.53 (1.36-1.71)
Mothers only	19,915	150,952	983 (651.2)	2.59 (2.43-2.76)	2.22 (2.08-2.36)	1.71 (1.60-1.83)
Both parents	398	2,732	37 (1354.1)	5.57 (4.04-7.69)	3.58 (2.60-4.94)	2.20 (1.59-3.05)

Abbreviations. ASD: Autism Spectrum Disorders.

Postpartum depression: any depression diagnosed in the first year after delivery.

Hazard Ratios with 95% confidence intervals from Cox regression models, using age at follow-up as the underlying time scale, with robust standard errors. Incidence rate of ASD per 100,000 person years. Followed up all children from age two.

Model 1: Adjusted for birth year by cubic natural splines with five internal knots;

Model 2: Adjusted for birth year, preterm birth (yes/no), maternal and paternal age (years; as natural cubic splines), yearly income (modelled by ranks as natural cubic splines), and highest educational attainment (< 9 years of primary education, 9 years of primary education, 1-2 years of secondary school education, 3 years of secondary school education, 1-2 years of postgraduate education, ≥ 3 years of postgraduate education, PhD education), all defined at delivery.

Model 3: Adjusted for birth year, preterm birth, maternal and paternal age, income, highest educational attainment, and any parental diagnosis of depression before delivery (yes/no).

eTable 7 Association between parental postpartum depression and offspring ASD risk among parents without an ASD diagnosis before delivery

Postpartum depression	Total subjects	Person-years	ASD (rate)	Model 1	Model 2	Model 3
Neither parent	1,751,676	21,431,773	49,671 (231.8)	Reference	Reference	Reference
Fathers only	6,252	56,005	330 (589.2)	2.56 (2.30-2.85)	1.88 (1.69-2.09)	1.53 (1.37-1.72)
Mothers only	21,360	171,297	982 (573.3)	2.59 (2.43-2.76)	2.22 (2.08-2.36)	1.71 (1.60-1.83)
Both parents	413	3,113	36 (1156.3)	5.44 (3.92-7.54)	3.50 (2.52-4.85)	2.15 (1.55-2.99)

Abbreviations. ASD: Autism Spectrum Disorders.

Postpartum depression: any depression diagnosed in the first year after delivery.

Hazard Ratios and 95% confidence intervals, as estimates of relative risk of ASD in offspring of parents with postpartum depression compared to offspring of parents without postpartum depression, were calculated from Cox regression models, using age at follow-up as the underlying time scale, with robust standard errors. Incidence rate of ASD per 100,000 person years. Followed up all children from age one.

Model 1: Adjusted for birth year by cubic natural splines with five internal knots;

Model 2: Adjusted for birth year, preterm birth (yes/no), maternal and paternal age (years; as natural cubic splines), yearly income (modelled by ranks as natural cubic splines), and highest educational attainment (< 9 years of primary education, 9 years of primary education, 1-2 years of secondary school education, 3 years of secondary school education, 1-2 years of postgraduate education, ≥ 3 years of postgraduate education, PhD education), all defined at delivery.

Model 3: Adjusted for birth year, preterm birth, maternal and paternal age, income, highest educational attainment, and any parental diagnosis of depression before delivery (yes/no).

eFigures

eFigure 1 Weighted Schoenfeld residual plot to assess proportional hazards assumption

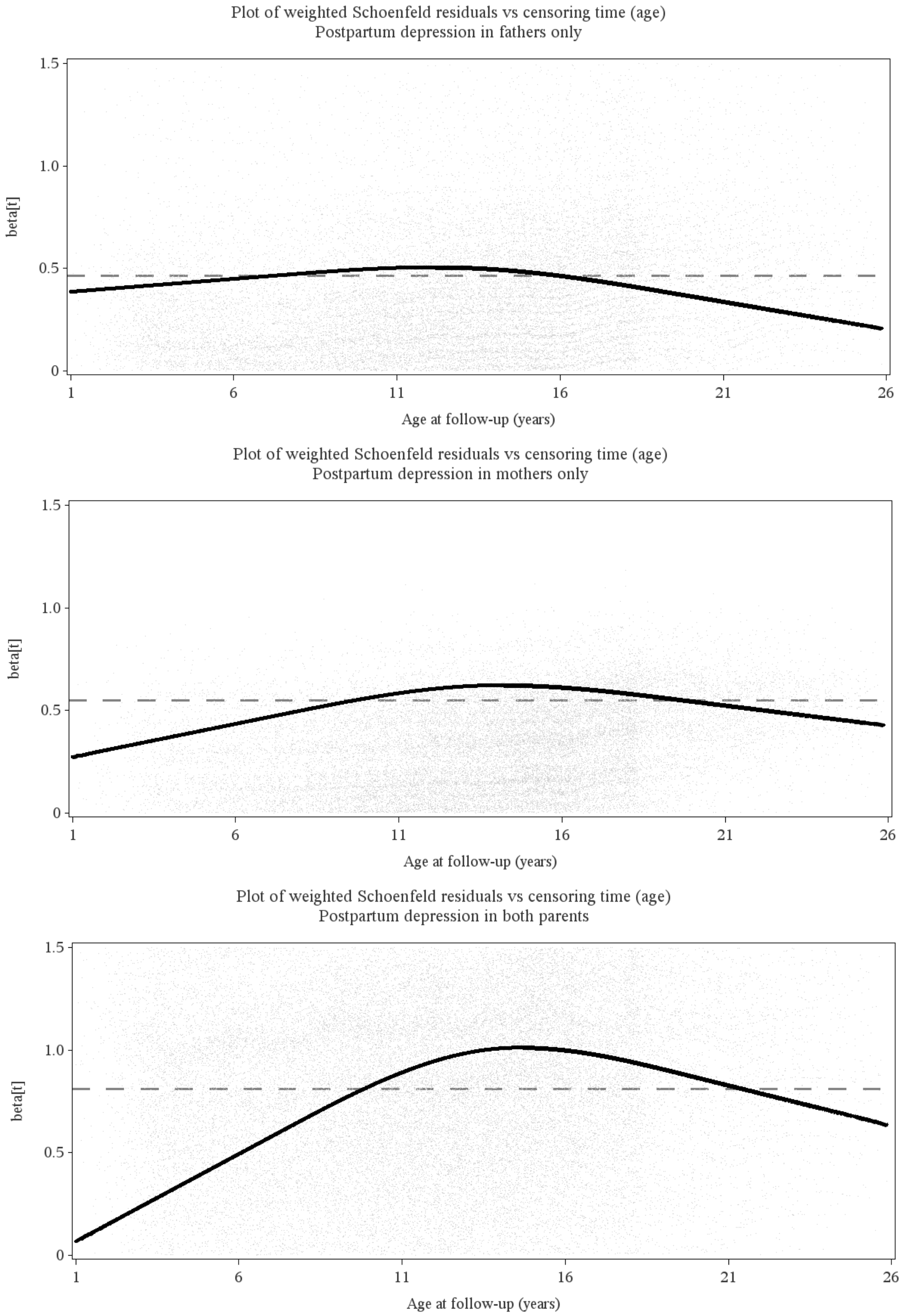

Horizontal dotted line correspond to mean residual which approximate the cox regression hazard ratio for the same parameter

Note: The proportional hazards assumption of the Cox regression was visually examined by Schoenfeld residuals, where a lack of systematic patterns or trends over time indicates adherence to the assumption. The figure displays age-specific *log hazard ratios* (beta=0 reprents hazard ratio=1) for the risk of ASD, adjusted for birth year, preterm birth, maternal and paternal age, income, highest educational attainment, and any parental diagnosis of depression before delivery. The age-specific hazard ratios are estimated by the weighted Schoenfeld residuals on the y-axis and x-axis, respectively. The graph displays a smoother that represents the average expected risk at each age.
